# AIP56: A Novel Bacterial Apoptogenic Toxin 

**DOI:** 10.3390/toxins2040905

**Published:** 2010-04-26

**Authors:** Manuel T. Silva, Nuno M. S. dos Santos, Ana do Vale

**Affiliations:** IBMC-Instituto de Biologia Molecular e Celular, Rua do Campo Alegre, 823. 4150-180 Porto, Portugal; Email: mtsilva@ibmc.up.pt (M.T.S.); nsantos@ibmc.up.pt (N.M.S.S.)

**Keywords:** *Photobacterium damselae* subsp. *piscicida*, AIP56, AB toxin, apoptosis, secondary necrosis

## Abstract

*Photobacterium damselae* subsp. *piscicida* (*Phdp*) is a Gram-negative pathogen agent of an important fish septicemia. The key virulence factor of *Phdp* is the plasmid-encoded exotoxin AIP56, which is secreted by exponentially growing pathogenic strains. AIP56 has 520 amino acids including an *N*-terminal cleavable signal peptide of 23 amino acid residues, two cysteine residues and a zinc-binding region signature HEXXH that is typical of most zinc metallopeptidases. AIP56 induces *in vitro* and *in vivo* selective apoptosis of fish macrophages and neutrophils through a caspase-3 dependent mechanism that also involves caspase-8 and -9. *In vivo*, the AIP56-induced phagocyte apoptosis progresses to secondary necrosis with release of cytotoxic phagocyte molecules including neutrophil elastase. Fish injected with recombinant AIP56 die with a pathology similar to that seen in the natural infection.

## 1. The Apoptogenic Exotoxin AIP56 Is the Key Virulence Factor of *Photobacterium damselae* subsp. *piscicida*

*Photobacterium damselae* subsp. *piscicida* (*Phdp*) is a Gram-negative pathogen that was isolated for the first time from a massive fish kill in Chesapeake Bay [1]. The disease has been named pseudotuberculosis and, after the initial classification of this agent as *Pasteurella piscicida* [2], as pasteurellosis. The pathogen was later reassigned to the genus *Photobacterium* as *Photobacterium* *damsela* subsp. *piscicida* [3], the name afterwards corrected to *Photobacterium damselae* subsp. *piscicida* [4]. Consequently, the disease is also referred to as photobacteriosis. *Phdp* is highly pathogenic for wild and cultured marine fish and has been found to affect more than 20 warm waterfish species worldwide, including sea bass, sole, yellowtail, gilt-head sea bream and turbot [5,6,7]. *Phdp* infection is now recognized as one of the most threatening bacterial diseases in mariculture worldwide due to its wide host range, massive mortality, ubiquitous geographical distribution, widespread antibiotic resistance and lack of efficient vaccines [8]. In its acute form, this disease has a septicemic nature and has a short course and very high mortality [5,7,9].

Early descriptions of the histopathology of *Phdp* infection and of the toxicity of *Phdp* extracellular products recognized the occurrence of cytotoxic alterations [6,10,11,12,13,14,15,16], but the cytological characterization of these alterations and the mechanism for their production were not elucidated. The re-evaluation of the interaction of virulent *Phdp* with fish phagocytes was undertaken in our laboratory using the intraperitoneal (i.p.) inoculation in sea bass. This analysis revealed that the cytopathology previously described in *Phdp* infection is due to pathogen-induced macrophage and neutrophil apoptotic death [17]. The virulence of *Phdp* strains from different geographical locations, including Portugal, Spain, Italy, Greece and Japan, was associated to the apoptogenic activity of the pathogen [17]. 

Different studies have identified several factors/mechanisms that could be involved in the virulence of *Phdp* [6,11,18]. However, the recognition that (i) systemic macrophage and neutrophil apoptosis was a marked feature of experimental *Phdp* infections; (ii) apoptosis of macrophages and neutrophils was observed after injection of virulent bacterial culture supernatants but not of UV-killed virulent bacteria; and (iii) the apoptogenic activity of culture supernatants was abolished by heat-treatment, led us to admit that one (or several) protein(s) secreted by virulent *Phdp* strains would be responsible for the apoptogenic activityof these bacteria [17].

Considering that infecting *Phdp* in the host would be metabolically active, we searched for the putative apoptogenic factor in supernatants of mid-exponential cultures of *Phdp* [19]. Surprisingly, this search revealed a major protein band in the electrophoretic profiles of those supernatants of virulent *Phdp* but not in nonvirulent strains. This finding was in contrast to previous studies suggesting the secretion of a highly complex mixture of different proteins by *Phdp* [20]. The larger protein complexity of the culture supernatants observed by those researchers could be explained by the fact that stationary-phase cultures were used in their studies. 

Concentrated cell-free culture supernatants from a virulent *Phdp* strain were resolved by native-PAGE, fractionated, and the fraction containing the major protein showed *in vivo* capacity to induce apoptosis of sea bass macrophages and neutrophils, pointing to the likelihood of it being the virulent apoptogenic factor of pathogenic *Phdp*. SDS-PAGE analysis of that fraction revealed a single protein band of approximately 56 kDa. The protein was named AIP56 (*a*poptosis *i*nducing *p*rotein of 56 kDa) [19].

Recombinant AIP56 expressed in *Escherichia coli* BL21 was found to retain the apoptogenic activity of *Phdp* AIP56 towards macrophages and neutrophils when injected i.p. in sea bass or when the toxin was incubated *ex vivo* with peritoneal sea bass phagocytes. These results demonstrated that the exotoxin AIP56 is the factor responsible for the apoptogenic activity of virulent *Phdp* [19].

The primary structure of AIP56 has been characterized [19]. AIP56 was annotated as a 513 amino acid precursor protein, including an N-terminal cleavable signal peptide of 16 amino acid residues (GeneBank Accession number DQ066884). However, an alternative GTG start codon exists 21 nucleotides ahead of the previously annotated ATG start codon, extending the signal peptide to 23 amino acids and the full length protein to 520 amino acids. Moreover, AIP56 displays a hydropathic profile typical of a non-membrane protein. 

**Table 1:** Sequences producing significant alignments from Blast analysis of the AIP56 protein sequence against the non-redundant protein database at http://blast.ncbi.nlm.nih.gov/Blast.cgi.

Accession	Description	Max score	Total score	Query coverage	E value
YP_003422532.1	Aip56 [*Photobacterium damselae* subsp. *piscicida*]	1064	1064	98%	0.0
BAF99004.1	apoptosis inducing protein [*Photobacterium damselae* subsp. *piscicida*]	1046	1046	98%	0.0
ZP_02194626.1	hypothetical protein 1103602000593_AND4_00648 [*Vibrio* sp. AND4]	597	597	99%	9.00E-169
CBA72068.1	non-LEE encoded type III effector C [*Arsenophonus nasoniae*]	306	306	94%	3.00E-81
CBA72300.1	apoptosis inducing protein [*Arsenophonus nasoniae*]	281	281	93%	1.00E-73
CBA76058.1	non-LEE encoded type III effector C [*Arsenophonus nasoniae*]	275	275	93%	7.00E-72
YP_002308522.1	hypothetical protein D [Bacteriophage APSE-2]	194	194	36%	2.00E-47
CBA74519.1	non-LEE encoded type III effector C [*Arsenophonus nasoniae*]	192	192	94%	8.00E-47
ZP_04620510.1	Non-LEE encoded type III effector C [*Yersinia aldovae* ATCC 35236]	178	178	48%	2.00E-42
NP_286533.1	hypothetical protein Z0986 [*Escherichia coli* O157:H7 EDL933]	178	178	50%	2.00E-42
YP_003229213.1	T3SS secreted effector NleC-like protein [*Escherichia coli* O26:H11 str. 11368]	178	178	50%	2.00E-42
YP_002328603.1	T3SS secreted effector NleC homolog [*Escherichia coli* O127:H6 str. E2348/69]	178	178	50%	2.00E-42
YP_003234807.1	T3SS secreted effector NleC-like protein [*Escherichia coli* O111:H- str. 11128]	177	177	50%	4.00E-42
YP_003234967.1	T3SS secreted effector NleC-like protein [*Escherichia coli* O111:H- str. 11128]	174	174	50%	2.00E-41
YP_003365223.1	T3SS effector protein NleC [*Citrobacter rodentium* ICC168]	173	173	48%	6.00E-41
ZP_03043710.1	non-LEE encoded type III effector C [*Escherichia coli* E22]	172	172	50%	7.00E-41
ZP_02576318.1	non-LEE encoded type III effector C [*Salmonella enterica* subsp. *enterica* serovar 4,[5],12:i:- str. CVM23701]	169	169	48%	9.00E-40
YP_001762636.1	hypothetical protein Swoo_4286 [*Shewanella woodyi* ATCC 51908]	83.2	83.2	28%	6.00E-14
ZP_03318593.1	hypothetical protein PROVALCAL_01527 [*Providencia alcalifaciens* DSM 30120]	38.9	38.9	25%	1.7

**Figure 1:**
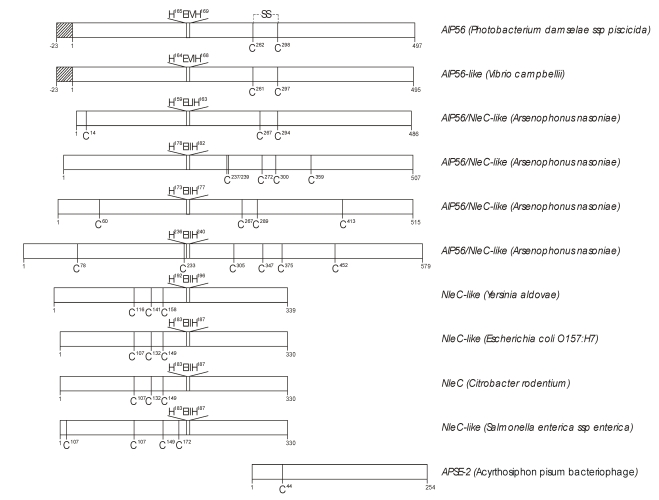
Schematic diagram comparing the main features of the primary structure of AIP56 with proteins retrieved by Blast analysis of the AIP56 protein sequence against the non-redundant protein sequences database. Striped bars represent signal peptides according to SignalP at http://www.cbs.dtu.dk/services/SignalP/ [26,27].The conserved zinc-metalloprotease signature HEXXH and cysteine residues are represented at their relative positions. Numbers represent amino acid residues.

Two other relevant aspects of AIP56 primary structure are the presence of only two cysteine residues (C^278^ and C^314^) and of a zinc-binding region signature HEXXH that is typical of most zinc metallopeptidases. Further characterization, using Blast analysis on non-redundant protein sequences database (http://blast.ncbi.nlm.nih.gov/Blast.cgi), revealed as the closest hit a homologous hypothetical protein of *Vibrio campbellii*, another widely distributed pathogen of cultured marine organisms [21] (Table 1). The next hits with the full length AIP56 were four recently annotated proteins of *Arsenophonus nasoniae*, a son-killer bacterium of the wasp *Nasonia vitripennis*, resulting from four open reading frames dispersed throughout the genome [22] (Table 1). These AIP56-like proteins present a primary structure highly similar to that of AIP56 (Figure 1). The Blast analysis also revealed the similarity of the N-terminal of AIP56 (first 324 amino-acids) with type III secreted effectors C from enteric pathogenic bacteria, and of the C-terminal with a hypothetical protein of the temperate lambda-like bacteriophage APSE-2, a phage that infects *Hamiltonella defense*, the endosymbiont of the pea aphid *Acyrthosiphon pisum* (reviewed in ref. [23]). Most remarkable is the conservation of the zinc-metalloprotease signature in the referred type III secreted effectors (Figure 1). The above mentioned analysis suggests that AIP56 has two domains, possibly linked by a disulfide bridge. This hypothesis has been supported in our laboratory by limited proteolysis experiments, which showed the existence of two major digestion fragments with sizes consistent with the two putative AIP56 domains predicted by homology analysis (unpublished results). N-terminal sequencing and MS analysis of the chymotrypsin digestion fragments revealed that the cleavage occurs between F^285^ and F^286^ lying in the amino-acid stretch between the two unique cysteine residues (C^278^ and C^314^), and analysis of the digests under non-reducing conditions confirmed the existence of a disulfide bridge linking the two proteolytic fragments, as a single band of 56 kDa was obtained. 

The above data led us to hypothesize that AIP56 is an AB-toxin, similar to tetanus and botulinum neurotoxins. Tetanus and botulinum neurotoxins are AB toxins that possess an A domain displaying metalloprotease activity linked to a B domain by a single disulfide bridge [24,25]. Considering that the AIP56 N-terminal region has a high identity with type III effectors from enteric pathogenic bacteria, and that a zinc-metalloprotease signature is conserved both in the AIP56 N-terminal and in the type III effectors, we hypothesize that the N-terminal region is responsible for the toxin’s apoptogenic activity and the C-terminal region would be responsible for the toxin binding/entry into the cells. Further studies are presently ongoing to assess this hypothesis.

As observed for many bacterial toxin-specific genes, which are often located on mobile genetic elements [28], AIP56 was also found to be encoded in a high-copy plasmid present in all virulent *Phdp* strains tested and from diverse geographical locations [19]. The location of the AIP56 gene in a high-copy plasmid is probably the reason why *Phdp* virulent strains are capable of producing large amounts of exotoxin.

Like in other pathogens, the virulence of *Phdp* certainly is multifactorial. However, the AIP56-dependent pathogenicity mechanism most likely is central in the etiopathogenesis of fish pasteurellosis because: (i) the AIP56 gene is present in all virulent strains and absent in the non-virulent isolates tested [19]; (ii) systemic phagocyte destruction similar to that seen in fish with advanced natural or experimental pasteurellosis was seen in moribund fish following the inoculation of recombinant AIP56 [29]; (iii) passive immunization with anti-AIP56 rabbit serum protects sea bass against *Phdp* infection [19].

The importance of this novel pathogenicity factor of *Phdp* is emphasized by the observation that AIP56-induced apoptotic phagocyte destruction observed in experimental infections also occurs in sea bass and sole with natural *Phdp* infection [29].

## 2. AIP56 Induces a Typical Apoptotic Death in Fish Macrophages and Neutrophils

**Figure 2:**
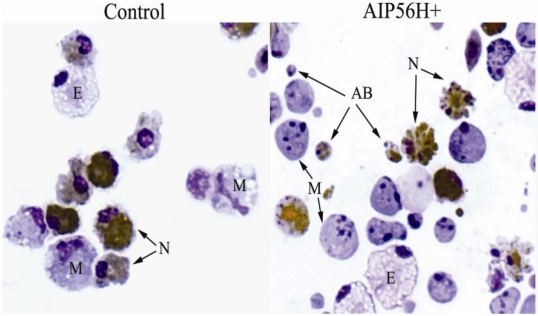
Wright-stained cytospins of sea bass peritoneal leukocytes 4 h after i.p. injection of 2 μg of recombinant AIP56 (AIP56H+) or the same amount of heat-inactivated recombinant AIP56 (control). Neutrophils were labeled by peroxidase detection (brown granules). In cells exposed to AIP56H+, note the occurrence of extensive nuclear fragmentation and chromatin condensation in macrophages (M) and neutrophils (N), cell blebbing in neutrophils, and the presence of apoptotic bodies (AB). The eosinophilic granular cells (E) look normal in both samples. Objective 100 ×. From reference [19].

The initial observations in sea bass experimental *Phdp* infection pointed to the selective targeting of both professional phagocytes by a *Phdp* cytotoxin [17]. To better dissect the anti-phagocyte apoptogenic activity of AIP56, an *ex vivo* model was used. The cells used in the *ex vivo* studies were inflammatory peritoneal leukocytes collected from peritoneal cavities of sea bass injected with Incomplete Freund’s Adjuvant 15 days before collection [19] (Figure 2). The apoptotic nature of the alterations seen in these leukocytes was established by the presence of a set of indicators [30,31,32]: cell shrinkage, chromatin condensation, nuclear fragmentation, production of apoptotic bodies, internucleosomal DNA degradation revealed by nuclear TUNEL positivity and by DNA electrophoresis, and activation of the apoptosis executioner caspase-3. These experiments showed that the only cells in the inflamed peritoneal population affected by the apoptogenic activity of AIP56 were macrophages and neutrophils [19]. The apoptogenic activity of AIP56 against the murine macrophage cell line J774, bone-marrow derived mouse macrophages and *Drosophila* S2 macrophage-like cell line was also tested. AIP56 did not display apoptogenic activity against those cells (unpublished results). 

Recent results [33,34] showed that the AIP56-induced selective apoptosis of fish macrophages and neutrophils also involves activation of caspases-8 and -9. Following the molecular cloning and characterization of sea bass caspases-3, -8, and -9, the expression of these caspases was analyzed in sea bass with experimental *Phdp* infection or injected with AIP56-containing *Phdp* culture supernatants. The very low basal expression of those caspases increased in the initial 12-24 h post-infection or post-injection. Additionally, caspase-3, -9, and -8 proteolytic activities were detected in organs of sea bass with terminal infection [29,33,34]. 

Involvement of mitochondria was also found, as shown by loss of mitochondrial membrane potential, translocation of cytochrome c to the cytosol and over-production of ROS.

## 3. Pathogenetic Consequences of the *in Vivo* Cytopathological Effects of AIP56 Toxin

Apoptosis [35] is one mode of active cell death that, in multicellular animals, is a major physiological cell killing process used during embryonic morphogenesis and in adult life in tissue homeostasis and immune responses for the elimination of unnecessary, unwanted or dangerous cells [36,37]. Typically, in physiological situations, the apoptotic mode of cell elimination is a useful process since it is induced when appropriate, comprises the induction of a safe mechanism for cell disposal through the removal of apoptosing cells by scavenger cells, and, although spending energy, is economical in terms of re-utilization of the products of this disposal [35]. However, the perspective of apoptosis as a useful process is restrictive, since this mode of cell elimination can be subverted, leading to harmful pathological situations. In this context, it has to be noted that fully developed apoptosis leads to necrotic cell disintegration. Indeed, soon after the proposal the term apoptosis [35], it was recognized that *in vitro* apoptosis proceeds to necrosis leading to cell disruption [38,39], a process called secondary necrosis [30]. However, in multicellular organisms, the apoptotic process comprises a mechanism for the removal and degradation of the apoptosing cell through phagocytosis by scavenger cells, mainly macrophages [40,41]. This removal occurs while the apoptosing cell still is enveloped by a membrane that retains potentially harmful components, thus preventing the progression to secondary necrosis. When insufficient removal of apoptosing cells occurs, secondary necrosis ensues leading to cell lysis, which is pathogenetic [42]. Insufficient clearance of apoptosing cells *in vivo* has been described in situations of massive apoptosis that overwhelms the available scavenging capacity [43,44,45], or when this capacity is directly impaired by deleterious effects on macrophages [46,47,48,49]. 

Apoptosis also turns pathological when the killed cells are necessary and functional, for example in degenerative disorders and in infectious diseases when pathogens destroy protective immune cells leading to evasion from host anti-microbial mechanisms [50,51,52,53].

In the early phase of infection, *Phdp* is seen within macrophages in natural [9] and experimental *Phdp* infection [13,29]. However, as the infection progresses, systemic dissemination of the pathogen occurs as indicated by the isolation of increasing numbers of bacteria from the blood and organs in fish experimentally infected with *Phdp* [29]. Systemic dissemination is accompanied by extensive extracellular bacterial multiplication as suggested by the observation of dense aggregates of extracellular bacteria (micro-colonies) in infected tissues. Extracellular *Phdp* are present in the spleen, headkidney, liver, gut lamina propria and blood, indicating that advanced *Phdp* infection is a septicemic situation. The septicemic spread of *Phdp* in the infected host is paralleled by the occurrence of AIP56 in the plasma [29].

**Figure 3:**
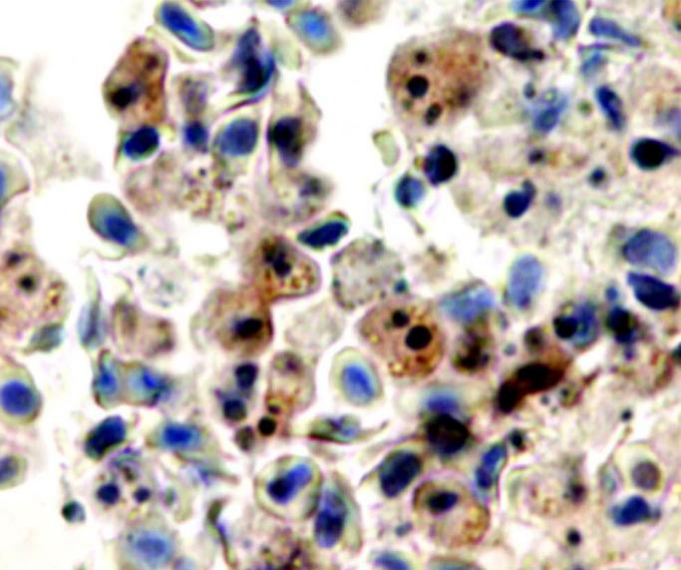
Blood in a vessel of the spleen of a sea bass with advanced natural *Phdp* infection labeled by immunocytochemistry for AIP56 exotoxin. Apoptosing cells with fragmented nuclei and/or condensed chromatin are immunostained for AIP56 (brown). Objective 100 ×. From ref. [29].

Extensive infiltration of macrophages and neutrophils occurs in the initial phase of infection when local multiplication of *Phdp* becomes detectable in infected tissues [29]. This initial accumulation of phagocytes is followed by extensive phagocyte depletion in advanced experimental *Phdp* infections [29]. This depletion correlates with the appearance of high numbers of macrophages and neutrophils with apoptotic features [17,29]. Apoptosing cells, identified by an apoptotic morphotype, TUNEL positivity and presence of active caspase-3, were seen in foci and scattered in the splenic and head kidney parenchymas, in the peripheral blood, in blood of the spleen, liver and head kidney vasculature and in gut lamina propria. In fish experimentally infected with *Phdp* or injected with the toxin AIP56, detachment of enterocytes was observed [54]; the detached enterocytes enter detachment-induced epithelial cell apoptosis or anoikis [55]. Supporting the interpretation that AIP56 is the apoptogenic factor of *Phdp* towards fish macrophages and neutrophils, leukocytes with apoptotic features present in *Phdp*-infected tissues immunostain for AIP56 [29] (Figure 3).

In advanced *Phdp* infection, foci of necrosis with abundant cell debris are prominent [29]. There are data that support the interpretation that this necrosis is the result of the progression of phagocyte apoptosis to secondary necrosis due to the lack of removal of abundant apoptosing phagocytes. Activated caspases are released by cells undergoing apoptotic secondary necrosis when rupture of the cytoplasmic membrane occurs [56] and occurrence of relevant apoptotic secondary necrosis can be detected *in vivo* by the presence of increased levels of extracellular activated caspases, which can be quantified in the blood [57,58,59]. An indication that the extensive phagocyte lysis in the advanced phase of the disease is due to the progression to secondary necrosis of the phagocytes is the observation in terminal infection of elevated blood levels of activated caspase-3 [29]. This result agrees with the observation by electron microscopy in fish with advanced *Phdp* infections of phagocytes with condensed chromatin and fragmented nuclei (typical of apoptosing cells) together with ruptured cytoplasmic membrane (typical of necrosis) [17], a hallmark of secondary necrosis [42].

Lysis of phagocytes by secondary necrosis, leading to the release of highly cytotoxic components, is particularly pathogenetic in the case of neutrophils. Neutrophils are extraordinarily rich in highly cytotoxic components which, if released in excess, can damage many types of cells, with the potential to produce tissue injury [60,61,62]. One such molecule is the destructive enzyme neutrophil elastase [62,63]. Active neutrophil elastase was found in the plasma of fish with terminal *Phdp* infection [29]. In fish with advanced disease the levels of circulating active neutrophil elastase highly correlated with the levels of circulating activated caspase-3, suggesting that in terminal *Phdp* infection neutrophil lysis was due to secondary necrosis.

Experimental [17,29] and natural [5,7] acute *Phdp* infections are rapid, severe infections with a very high mortality. This may be related to the characteristics of the AIP56-dependent *Phdp* pathogenicity mechanism that indicate that it is a highly potent one, as follows. First, using an exotoxin, this mechanism can operate at a distance without requiring contact between the pathogen and the target cells. Second, apoptotic destruction of both macrophages and neutrophils, AIP56 not only impairs the participation of these leukocytes in phagocytosis and killing of *Phdp*, but also releases intraphagocytic bacteria present in the initial phase of the infection, two effects that promote survival of the pathogen and its unrestricted extracellular multiplication. Concomitantly, the AIP56-induced apoptosis of both professional phagocytes leads to tissue damage with deleterious consequence for the host. In fact, destruction of macrophages, the cells with the crucial role of eliminating apoptotic cells [40,41], results in deficient clearance of AIP56-induced abundant apoptotic neutrophils, which leads to their lysis by secondary necrosis [17], with the severe consequences due to the release of highly cytotoxic neutrophil molecules [60,61,62]. Finally, relying on a potent protein exotoxin, this pathogenicity mechanism requires the production of anti-AIP56 antibodies as a defensive response from the host, an adaptive immune response that cannot be achieved in the short time lapse of this fulminant infectious disease.

In innate and adaptive anti-bacterial immune responses macrophages and neutrophils work in concert as crucial elements of host defense against infection as the effectors of the myeloid phagocyte system [64,65]. The ability of bacterial pathogens to cause timely phagocyte cell death is important for virulence [66], and this anti-phagocytic mechanism may follow phagocytosis of the pathogen, depend on direct contact between the pathogen and the host cell target with transfer of effector molecules by secretion systems [67,68], or be mediated by exotoxins as in the case of *Phdp*. The simultaneous targeting of macrophages and neutrophils by *Phdp* through the AIP56 exotoxin thus represents a very effective pathogenicity strategy which contributes to the severity of acute *Phdp* infections. This ability of pathogens to simultaneously target the two professional phagocytes *in vivo* is not common, but a few examples have been reported, as is the case of *Pseudomonas aeruginosa* [69,70], *Yersinia pestis* [71] and *Francisella tularensis* [72,73].

In conclusion, the identification, characterization and production of recombinant active AIP56 allowed the clarification of the key virulence factor of an important fish pathogen and revealed a relevant bacterial pathogenicity weapon relying on a largely underestimated anti-phagocyte mechanism based on the lysis by apoptotic secondary necrosis of macrophages and neutrophils by a bacterial cytotoxin. The observation that neutralization of AIP56 activity by transfer of anti-AIP56 antibodies is effective in protecting sea bass against experimental *Phdp* infection open promising prospects towards the development of an effective anti-*Phdp* vaccine based on AIP56. Finally, the observed homology between AIP56 and type III secreted effectors from enteric pathogenic bacteria suggests possible commonalities between pathogenicity mechanisms of *Phdp* and those enteropathogens, with implications for human infections. 
